# Foot temperature responses during walking: a theoretical estimation of mechanical and physiological factors

**DOI:** 10.3389/fbioe.2025.1628846

**Published:** 2025-11-20

**Authors:** Jenna K. Burnett, Jose G. Anguiano-Hernandez, Kota Z. Takahashi

**Affiliations:** 1 Department of Health and Kinesiology, Sayu Lab for Biomechanics and Locomotion, University of Utah, Salt Lake City, UT, United States; 2 Department of Physical Medicine and Rehabilitation, University of Utah, Salt Lake City, UT, United States; 3 Department of Biomedical Engineering, University of Utah, Salt Lake City, UT, United States

**Keywords:** skin temperature, foot mechanics, blood flow, thermoregulation, thermodynamics, long distance walking, skin injury

## Abstract

Skin temperature has been previously associated with tissue complications (i.e., foot ulcers) in individuals with diabetes. Previous studies have suggested that mechanical factors during ground contact (i.e., foot shear force, foot net work), and physiological factors (i.e., skin blood flow) contribute to foot temperature changes during activities such as walking. However, it is currently unclear how mechanical and physiological factors interact to influence foot temperature. Therefore, the goal of this study was to present a framework to generate new hypotheses about the factors that drive foot temperature responses in healthy young adults during walking. This goal was accomplished in a two-part study. In the first part of the study, we collected experimental temperature data on the foot of 8 healthy young adults (1F/7M) during a 30-min walk. We then modeled the temporal response of temperature data using a linear regression and a non-linear logistic model. We reasoned that if mechanical factors alone influence the temperature responses, then the temperature would increase linearly over the walking duration (assuming similar foot mechanics from step-to-step). On the other hand, if physiological factors were at play, then the temperature would increase non-linearly over the walking duration. Experimental data indicated that the whole foot temperature increased by 3.50 ± 2.38 °C after 30 min of walking. Furthermore, the non-linear model better captured the temporal response (compared to linear model), which likely hints that both mechanics and physiology influence foot temperature. In the second part of the study, we applied a theoretical thermodynamics computation to parse the contributions of mechanical and physiological factors during walking. This computation predicted that mechanical factors accounted for 2.5 °C of the increase in foot temperature after 30 min of walking (out of the 3.5 °C), while physiological factor accounted for 1.0 °C. Furthermore, the physiological factors displayed a non-linear response during the walking duration, which were qualitatively similar to published reports of skin temperature outside the foot. Altogether, our study provides new hypotheses regarding the interaction between mechanical and physiological factors involved in foot temperature regulation, and may provide a framework to study populations at risk for foot complications due to impaired temperature regulation.

## 1 Introduction

Foot temperature has been found to be associated with foot ulcer development in individuals with diabetes (Ena et al., 2021; Yavuz et al., 2019; van Netten et al., 2014). These ulcers, if untreated, may eventually cause further complications such as amputation (Mifsud et al., 2022). However, temperature regulation of the skin in these clinical populations is not well understood, despite the evidence that foot temperature regulation may be imperative for skin protection. There is a critical need to develop new experimental frameworks to understand factors, including mechanical and physiological, that contribute to normal and abnormal foot temperature regulation.

Previous research (Papachatzis et al., 2022; Yavuz et al., 2014; Gonzalez et al., 2021) has suggested foot mechanics and mechanical work as potential contributors to foot temperature changes during activity. For example, during a single stride cycle, the foot experiences mechanical work or factors such as deformation (Wearing et al., 2014), energy dissipation (Papachatzis et al., 2022; Papachatzis and Takahashi, 2023; Zelik and Honert, 2018; Takahashi et al., 2017; Farris et al., 2019), and shear forces (Yavuz et al., 2014; Gonzalez et al., 2021) in response to ground contact. Because the foot is expected to return to approximately the same energy state at the end of the stride cycle, any work done during the stride is likely transformed into heat by thermodynamics. The generated heat would then be expected to lead to temperature changes on the foot’s surface which would accumulate throughout activity. However, the heat generated by mechanical factors and work during a stride cycle may not be the only sources of foot temperature change during activity.

Physiological factors such as blood flow, sweating, and cooling mechanisms (conduction, convection) from the skin surface may be additional sources of skin temperature change at the feet during activity. For example, blood flow through capillaries and arterio-venous anastomoses (AVAs) has been found to be associated with skin temperature changes at the feet and hands during activity (Tanda, 2015; Xu et al., 2008; Sagaidachnyi et al., 2019; Bornmyr et al., 1997; Taylor et al., 2014) and exposure to cold environments (Singh et al., 2024). As blood flow to the feet changes via capillaries and AVAs during activity or in response to the environment temperature, the skin temperature would be expected to also change due to the passive transfer of heat from the blood to surface of the skin (Bornmyr et al., 1997; Singh et al., 2024; Wang et al., 2022; Caldwell and Taylor, 2014). Similarly, other physiological responses to activity such as heat loss and cooling via conduction (Bornmyr et al., 1997; Arens and Zhang, 2006), sweat evaporation (Arens and Zhang, 2006; Machado-Moreira et al., 2008; B and aker, 2019), and convection (Arens and Zhang, 2006; De Dear et al., 1997; Oguro et al., 2002) have been found to influence the skin temperature. The combination of the physiological factors likely results in net heat generation or loss throughout activity which may contribute to the temperature changes at the surface of the skin.

It is currently unclear how the physiological and mechanical factors may interact at the feet to result in temperature change during activity. Previous research generally studied either mechanics (Papachatzis et al., 2022; Yavuz et al., 2014) or physiology (Tanda, 2015; Xu et al., 2008; Sagaidachnyi et al., 2019; Bornmyr et al., 1997; Taylor et al., 2014) in isolation, and have not explored these factors at the feet simultaneously (Nemati et al., 2021). Understanding how these factors (mechanical, physiological) interact may be important for providing clinical recommendations and focused interventions which address the relationship between foot temperature and skin breakdown in clinical populations.

One potential way to discern how mechanical and physiological factors contribute to foot temperature during activity is to characterize the temporal temperature response during a long walk, coupled with additional theoretical reasoning. We speculate that the temporal pattern of the foot temperature during a long walk (i.e., linear or non-linear response) could indirectly suggest which factors (mechanical or physiological, or both) are driving the temperature changes. For example, if mechanical factors drive the foot’s temperature response during a walk, the heat generated by the mechanical factors would be expected to be constant from stride to stride (i.e., linear increase in foot temperature during the walk). However, if physiological factors contribute to the foot’s temperature response, the heat which is lost through thermoregulatory mechanisms at the skin’s surface such as skin blood flow and sweating would be expected to vary during the walk. Therefore, we expect this variance in physiological factors to result in a non-linear temperature response throughout the walk. Characterizing the experimental temperature response during a long walk may provide an insightful dataset and a new framework to study the complex interactions of mechanical and physiological factors that affect foot temperature regulation.

The overall goal of this study was to generate new hypotheses regarding the mechanisms (mechanical and physiological) that drive foot temperature response in healthy adults during walking. We aimed to address this goal in two ways. First, we collected experimental foot temperature data in healthy adults during a 30-min walk. We then fit the temperature data using linear or non-linear model, to suggest whether mechanical factors alone (linear model) or a combination of mechanical and physiological factors (non-linear model) underly foot temperature changes. Second, to pose new hypotheses parsing the individual contributions of mechanical and physiological factors, we used a theoretical thermodynamics computation. Our proposed theoretical model is not intended predict actual foot temperature changes during a walk. Rather, the intent is to generate new hypotheses for the complex mechanisms that underlie healthy foot temperature regulation, which may suggest a theoretical framework to study impaired temperature regulation in diabetic feet.

## 2 Experimental methods and results

### 2.1 Experimental methods

**FIGURE 1:**
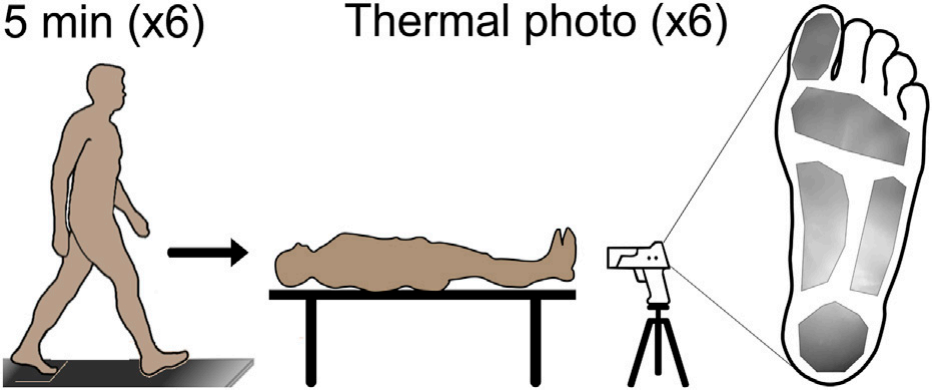
Prior to walking, participants lay supine for 15 min to allow the temperature to reach equilibrium; then a thermal photo was taken for the baseline foot temperature. After the baseline photo, the participants began the 30-min walk overground at 1.25 m/s, with the walk paused every 5 min for a thermal photo to be taken. A total of six photos were taken during the walk for analysis. Manual segmentation of the foot to identify the plantar hallux, metatarsophalangeal joint, midfoot, arch, and heel regions was completed, and the average regional foot temperature was extracted for analysis. A representative segment thermal photo is shown on the right side of the figure.

Eight healthy adults (1F/7M, 27.14 ± 3.53 years old, 80.42 ± 13.67 kg, 1.72 ± 0.05 m) were recruited for this study. Participants entered the lab and lay supine for 15 min. After 15 min, their shoes were removed, and a thermal photo (FLIR T540sc, Teledyne FLIR, Wilsonville, Oregon) was taken of the bottom of their feet. Participants then put their shoes back on and walked overground at 1.25 m/s for a total of 30 min. Walking speed during the 30-min walk was controlled by a trained pacer who walked alongside the participants. During the 30-min walk, the participants paused every 5 minutes for a thermal photo. During each pause, the participants lay supine with their shoes removed so a thermal photo could be taken of the bottom of their feet. The thermal photo of the foot at each time point was manually segmented to identify the hallux, metatarsophalangeal (MTP) joint, midfoot, arch, and heel. The average temperature in each region within each thermal photo was extracted for temporal analysis (Figure 1).

**FIGURE 2:**
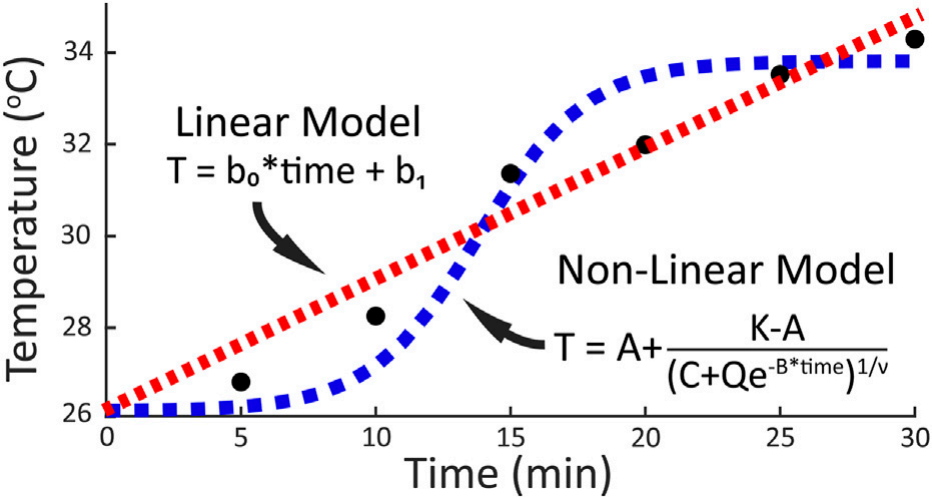
Prospective models and the respective general equations fit to temporal temperature change during a 30-min walk. A linear relationship (red line) between temperature and activity time would be expected if mechanical factors drive the temperature response. A non-linear relationship (blue line) between temperature and activity time would be expected if physiological factors contribute to the temperature response.

To determine how the foot temperature changed during walking, the temporal data for each foot region were fit with two different models: a linear regression equation, and a non-linear general logistic equation. A linear regression equation was chosen for the linear model as it was expected that mechanical factors would result in an iterative temperature response where temperature increases by a unit amount during each stance phase. A general logistic equation was chosen for the non-linear model as previous research has suggested that physiological factors may result in a S-shaped temperature response where temperature initially decreases, then increases and plateaus during running (Tanda, 2015). Examples of both models and their hypothesized fit to the data are shown in Figure 2. For the fitting procedure, custom equations of the forms T=m*time+b and $T=A+\frac{K-A}{{\left(C+Qe^{-B*time}\right)}^{1/\nu }}$ were fit using the fitlm or fitnlm functions within Matlab. The Matlab functions then output the coefficients (2 for the linear model, 6 for the non-linear model) which provide the best fit to the temporal data based on QR decomposition for the linear model or Levenberg-Marquardt nonlinear least squares algorithm for the non-linear model. To assess fit, the mean squared error (MSE) and residual standard deviation (S_res_) were chosen for comparison between models, as the traditional *R* ^2^ value is not considered a valid criteria when evaluating non-linear equation fit (Spiess and Neumeyer, 2010). The MSE and S_res_ assess the difference between the fitted data points and the measured data points, therefore, lower values of both are associated with models that more accurately predict the experimental data.

To calculate each participant’s whole foot temperature (***T***_***FootExp***_ ***(time)***) at each 5-min time point, a weighted average equation based on the regional and total surface area was used. The weighted average equation (Equation 1) multiplied the regional temperature at a specific time point (***T***_***region***_ ***(time***) by the region’s surface area within the thermal image at the respective time point (***A***_***region***_ ***(time)***). Region in this case refers individually to the hallux, MTP, midfoot, arch, and heel respectively. The weighted regional temperature was then summed across regions and then divided by the total surface area of the regions (***A***_***Total***_ ***(time)***) at that time point.

$$T_{FootExp}\left(time\right)=\frac{\sum A_{region}\left(time\right)*T_{region}\left(time\right)}{A_{Total}\left(time\right)}$$ (1)

The average whole foot temperature at each 5-min time point during the walk was then calculated across the participants using the weighted average whole foot temperature. The change in whole foot temperature during the 30-min walk (**ΔT**_**FootExp**_) was calculated as the difference between the whole foot temperatures at baseline and the end of the walk.

### 2.2 Experimental results

**FIGURE 3:**
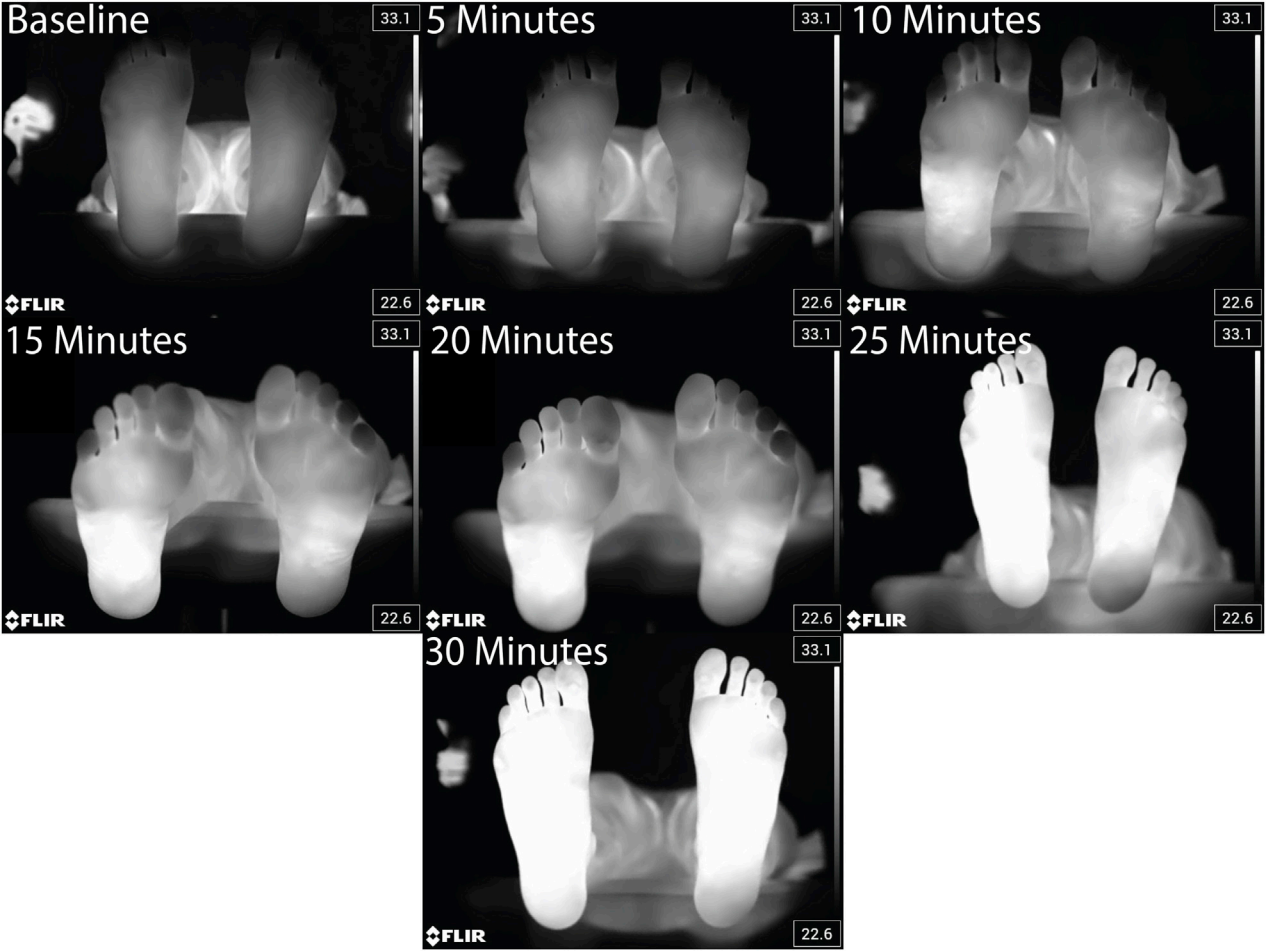
Representative thermal images taken every 5 min before, during, and at the end of the 30 min walk for a single participant.

**FIGURE 4:**
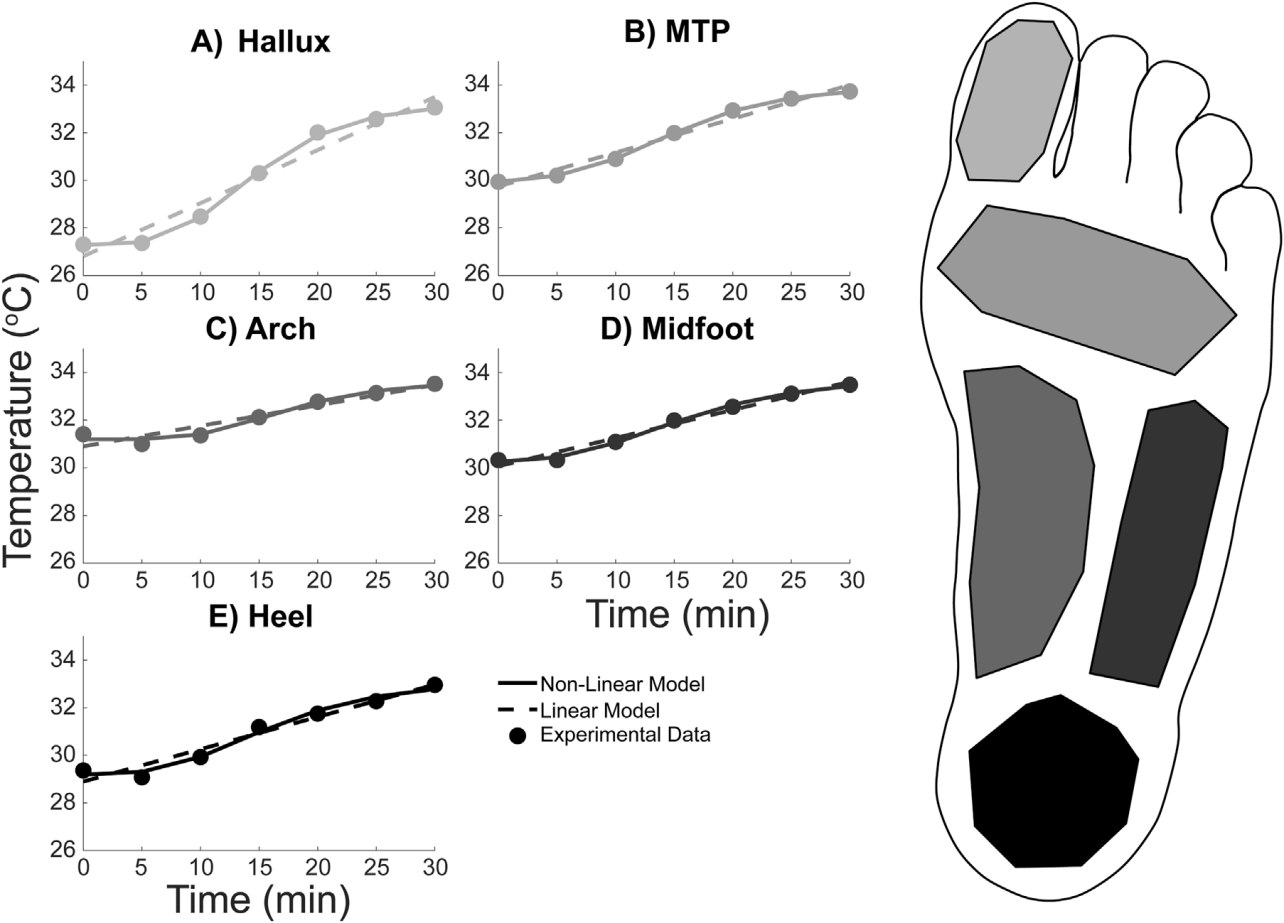
Average foot temperature in five regions during a 30-min walk in healthy adults: **(A)** Hallux, **(B)** Metatarsal-phalangeal (MTP), **(C)** Midfoot, **(D)** Lateral arch, and **(E)** Heel. The experimental temperature data (data points) was extracted from thermal photos taken every 5 min of the walk. The temperature data points were then fit with two equations: a linear regression equation (dashed line) and a non-linear logistic equation (solid line). Line and data point colors in the figure correspond to the foot region highlighted on the right.

Foot temperature (Figures 3, 4) in healthy adults during a 30-min walk increased by between approximately 2 and 6 °C, depending on the region (Hallux: 5.76 ± 3.79 °C; MTP: 3.80 ± 2.83 °C; Arch: 2.11 ± 1.90 °C; Midfoot: 3.15 ± 1.97 °C; Heel: 3.60 ± 2.29 °C) (Figure 4). When averaged across the whole foot using Equation 1, the whole foot temperature was found to increase by 3.50 ± 2.38 °C.

The temporal temperature response during the walk for each foot region was fit with the linear and non-linear equations (Figure 4). Equation coefficients which were output from the fitting procedure are contained within Table 1, 2 for the linear and non-linear equations respectively. The non-linear equation resulted in a lower MSE and S_res_ for all foot regions (Table 3). Therefore, the non-linear model is likely a more accurate prediction of the foot temperature than the linear model. The improved fit with the non-linear model, compared to the linear model, suggests that the mechanical and physiological factors may both contribute to foot temperature changes during walking in healthy adults.

**TABLE 1:** Coefficients for the linear equation ( T=m*time+b ) output from the fitting procedure.

Foot region	m	b
Hallux	0.22	26.81
MTP	0.14	29.74
Arch	0.09	30.90
Midfoot	0.12	30.07
Heel	0.14	28.90

**TABLE 2:** Coefficients for the non-linear equation ( $T=A+\frac{K-A}{{\left(C+Qe^{-B*time}\right)}^{1/\nu }}$ ) output from the fitting procedure.

Foot region	A	K	C	Q	B	ν
Hallux	27.28	32.80	0.994	0.92	0.19	0.078
MTP	29.88	33.69	0.976	4.79	0.18	0.43
Arch	31.20	31.61	0.998	0.017	0.17	0.0012
Midfoot	30.25	31.50	0.999	0.0081	0.14	0.0014
Heel	29.19	30.11	0.999	0.0057	0.15	0.00078

**TABLE 3:** Equation fit criteria (S_res_, MSE) for the linear and non-linear model at each temperature measurement location.

		Hallux	MTP	Midfoot	Arch	Heel
Residual Standard Deviation (S_res_)	Linear Model	0.579	0.283	0.337	0.231	0.363
	Non-Linear Model	0.084	0.016	0.146	0.086	0.208
Mean Squared Error (MSE)	Linear Model	0.335	0.08	0.113	0.053	0.132
	Non-Linear Model	0.018	0.001	0.053	0.018	0.108

## 3 Theoretical methods and results

Based on the experimental non-linear temperature response, the foot’s temperature change is theorized to be a summation of the individual temperature changes contributed by the mechanical and physiological factors. Because the mechanical factors (e.g., foot net work) are expected to remain relatively constant from step-to-step during the walking activity, the temperature change contributed by these factors is expected to be linear. However, because the physiological factors (e.g., blood flow to the skin, sweat production and evaporation, conduction, convection) are expected to vary throughout the activity (Johnson and Rowell, 1975; Kellogg et al., 1991a; Kellogg et al., 1991b; Johnson et al., 2010; Kenney and Johnson, 1992; B et al., 1977), the temperature change contributed by these factors is expected to be non-linear. Therefore, the summation of the linear contribution by the mechanical factors (Δ ***T***_***Mechanical***_ ***(time)***) and the non-linear contribution by the physiological factors (Δ ***T***_***Physiological***_ ***(time)***) is expected to result in the overall non-linear experimental temperature change (Δ ***T***_***FootExp***_ ***(time)***) during the walking activity.

$${\Delta T}_{FootExp}\left(time\right)={\Delta T}_{Mechanical}\left(time\right)+\Delta T_{Physiological}\left(time\right)$$ (2)

Therefore, to explore the potential contributions by each factor, a thought exercise that estimates the mechanical and physiological contributions to the temperature change was conducted. A theoretical thermodynamics model which relates foot work and temperature change was used to estimate the theoretical mechanical contribution to the experimental temperature change. Any differences between the experimental temperature and the mechanical factor’s theoretical temperature change were then hypothesized to be contributed by the physiological factors.

### 3.1 Theoretical methods

#### 3.1.1 Estimated temperature change from mechanical factors

For this thought exercise, the theoretical thermodynamics model is predicated on the First Law of Thermodynamics and the assumption that no energy is gained or lost during a single stride. In other words, because the foot is expected to be at same mechanical state at the beginning and end of a stride, the net energy of the stride will be zero. Because the net energy is zero, the net work done by the foot would be expected to be equal to the heat which is generated during the stride. Because work and heat are equal, work can replace heat in the equation which equates heat to whole foot temperature change during a single stride (Equation 3).

$$\Delta T_{stride}=\frac{-W_{stride}}{m_{foot}*c_{foot}}$$ (3)

In order to estimate the change in whole foot temperature due to the mechanical factors during a single stride (***ΔT***_***stride***_ ***,*** Equation 3), the work, foot mass, and specific heat capacity constant of the foot can be estimated based on the literature. First, the mass-normalized net work (Papachatzis et al., 2020) done during a single stride (N = 21 (6F/15M), Age: 24.14 ± 2.88 years, Height: 173.61 ± 6.67 cm, Mass: 83.17 ± 20.26 kg, Walking Speed: 1.25 m/s) was multiplied by this study’s average participant mass (80.42 ± 13.67 kg) to estimate stride net foot work (***W***_***stride***_ *,* Table 4). The foot mass (***m***_***foot***_ *,* Table 4) was based on standardized segment anthropometry (foot mass: 0.0145*body mass) (Winter 1990) multiplied by this study’s experimental average mass (80.42 ± 13.67 kg). The whole foot specific heat capacity constant (***c***_***foot***_ *,* Table 4) was estimated based on a weighted average of the material properties of the foot tissue (Taylor et al., 2014; Xu et al., 2023). The specific heat capacity constant represents the thermal energy that must be input to a material to increase the temperature one degree and varies with material properties. A more detailed discussion of how the whole foot specific heat capacity constant was calculated is included in the Supplementary Material. To get the temperature change during a single stride (Equation 3), the estimated stride net work was divided by the foot mass and the specific heat capacity constant. To estimate the total temperature change during the 30 min walk from mechanical factors (***ΔT***_***Mechanical***_ ***(time),*** Equation 4), the stride temperature change was multiplied by the average number of strides taken by our experimental cohort throughout the 30 min walk (***strides(time)*** *,* Table 4; Fra et al., 2012).

$$\Delta T_{Mechanical}\left(time\right)=\Delta T_{stride}*strides\left(time\right)$$ (4)

**TABLE 4:** Variables used as inputs to the theoretical thermodynamics framework to estimate the foot’s temperature change from mechanical factors.

Variable	Description	Mean value	Upper and lower bounds	References
*W*_*foot*_	Estimated net work done by the foot during a single stride	−0.0034 kJ	(-0.0071, 0.00023)	Papachatzis et al. (2020)
*strides*	Number of strides during 30 min of walking	1,674	-	Fra et al. (2012)
*m*_*foot*_	Foot mass	1.17 kg	-	Winter (1990)
*c*_*foot*_	Specific heat constant	1.96 kJ/K	(1.31, 3.62)	Taylor et al. (2014); Xu et al. (2023)

The above calculation was repeated using an upper and lower bound for net work and specific heat constants. Using the upper and lower bounds of net work and specific heat capacity constant characterized the possible range of temperature responses from mechanical factors. The upper and lower bounds for net work (Table 4) were calculated by adding or subtracting one standard deviation from the average net work in (Papachatzis et al., 2020). The upper and lower bounds for the specific heat constants (Table 4) were chosen based on the tissue that conducts heat the best (blood) and conducts heat the worst (bone) (Xu et al., 2023).

#### 3.1.2 Estimated temperature change from physiological factors

After estimating the mechanical factor’s contribution to the foot experimental temperature using the thermodynamics model, the estimated temperature from the physiological factors (***ΔT***_***physiological***_ ***(time)***) can be estimated by re-arranging Equation 2. In other words, the physiological factor’s contribution Equation 5 is the difference between the experimental temperature change (***ΔT***_***FootExp***_ ***(time)***) and the estimated mechanical factor’s temperature change (***ΔT***_***Mechanical***_ ***(time)***).

$${\Delta T}_{Physiological}\left(time\right)={\Delta T}_{FootExp}\left(time\right)-{\Delta T}_{Mechanical}\left(time\right)$$ (5)

#### 3.1.3 Estimated time to reach a first degree burn threshold

As an additional theoretical calculation and to contextualize the results, the extrapolated walking time required for the foot to surpass the temperature where first degree burns (>43.3 °C) (Johnson et al., 1974; Moritz and Henriques, 1947) may occur was also estimated. While surpassing this threshold is not a guarantee that first degree burns will occur, exposing the skin to temperatures beyond this threshold for an extended period may lead to first degree burns over time (Moritz and Henriques, 1947). More complex thermal damage models are available (e.g., Arrhenius) (Singh, 2022a), but these more complex damage models are beyond the scope of this study. Therefore, the simplified temperature threshold commonly associated with first degree burns was chosen to characterize whether the mechanical factors experienced by the foot during a long walk (i.e., over 3 hours) may result in first degree burns and potentially further indicate the importance of physiology in providing protection from injury. The change in temperature based on the estimated mechanical factors was calculated for a 3-h walk and then added to the average whole-foot experimental temperature. The walking time where the temperature surpassed 43.3 °C was then identified.

### 3.2 Theoretical results

#### 3.2.1 Estimated temperature change from mechanical factors

**FIGURE 5:**
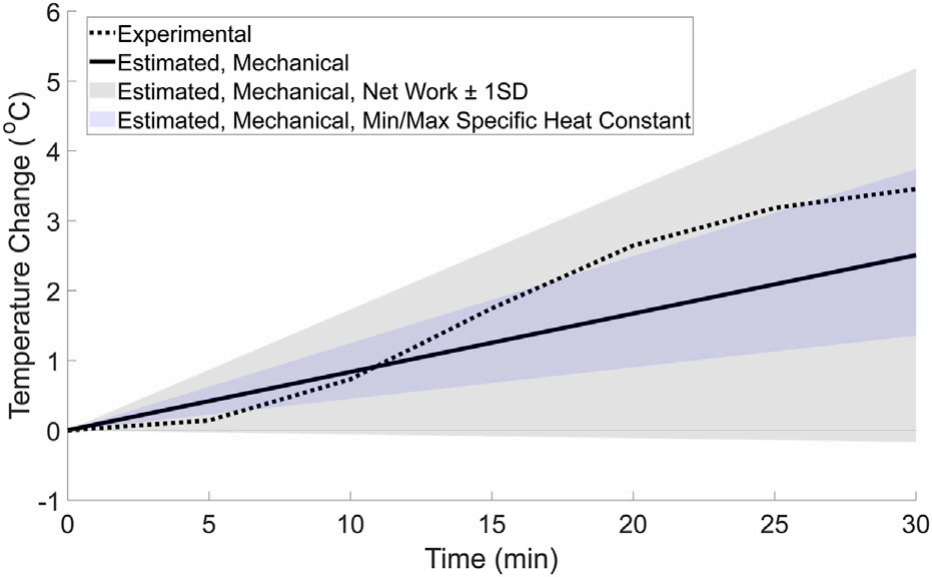
Estimated whole-foot temperature change from mechanical factors based on the basic thermodynamic framework (solid line). For comparison, the range of possible temperatures for the mechanical factor’s contribution were calculated using net work ±1 standard deviation (gray shaded region), and the minimum and maximum values for specific heat constants (blue shaded region). The whole foot’s experimental temperature change (dotted line) was calculated for comparison using a weighted average equation and is displayed in the figure.

Using the thermodynamic framework, the mechanical factor’s contribution to the experimental temperature change was estimated to be a linear increase in temperature of approximately 2.5 °C over the 30-min walk. When comparing this to the whole foot’s experimental temperature change, the mechanical factor’s contribution (Figure 5, solid line) is initially greater than the experimental temperature change during the 30-min walk (Figure 5, dashed line). However, after approximately 12 min, the experimental temperature surpasses the estimated temperature change contributed by the mechanical factors. Therefore, at the end of the 30-min walk, the temperature change from the mechanical factors alone is lower than the experimental temperature change of 3.50 °C. In addition, the experimental temperature is contained within the range of possible temperatures estimated using the upper and lower bounds for the foot’s net work in the thermodynamic estimation (Figure 5, gray shaded region), but not fully in the range estimated using the upper and lower bounds for the specific heat constant. This indicates that the experimental temperature change may be possible for a variety of mechanical factor magnitudes and a relatively large range of tissue distributions within the foot. However, it is worth noting that the temperature range estimated using the average net work ± one standard deviation (Papachatzis et al., 2020) has a lower bound that results in a small negative temperature change. This lower bound is likely the result of one participant in the data set used for the net work, as this participant’s foot work was an outlier that resulted in a large standard deviation value.

#### 3.2.2 Estimated temperature change from physiological factors

**FIGURE 6:**
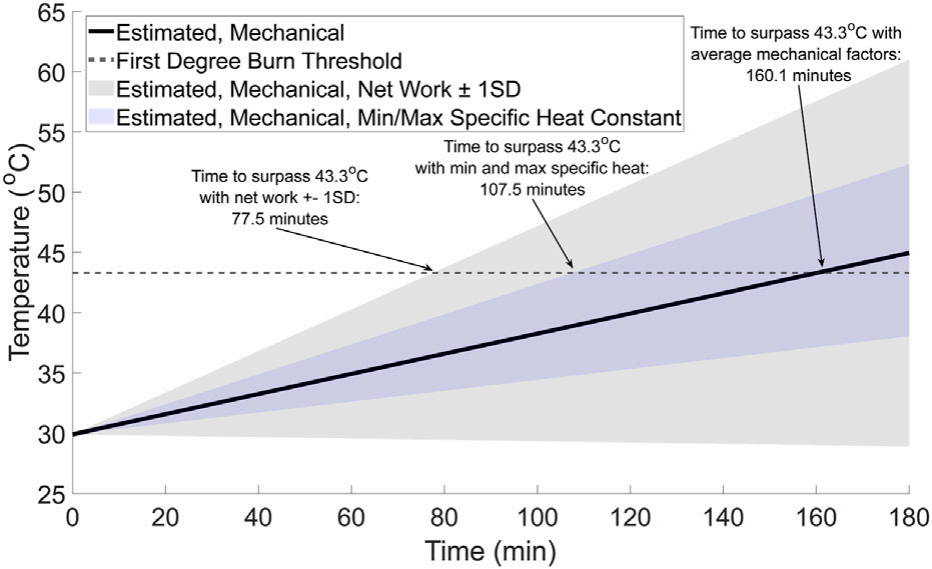
Estimated walking time to surpass first degree burns temperature (gray dashed line) from potential mechanical factors (black solid line). For comparison, the range of possible temperatures from the mechanical factors was calculated using the estimated upper and lower bounds for the mechanical factors based on the net work ±1 standard deviation (gray shaded region) and the minimum and maximum specific heat constants (blue shaded region).

The difference between the experimental temperature change and the estimated mechanical factor’s temperature change is hypothesized to be due to the net heat generated by physiological factors such as capillary and AVAs blood flow, conduction, convection, and sweat evaporation. The estimated contribution from the physiological factors (Figure 6, solid line) resulted in a non-linear change in temperature. This non-linear change in temperature appears similar to the temperature change across other regions of the body that do not experience high magnitude mechanical factors during running (Tanda, 2015), where there is an initial decrease in temperature, followed by an increase, and then plateau. Similarly, when the potential range of temperatures contributed by physiological factors is estimated by subtracting the upper and lower bounds of temperature change from the mechanical factors, the possible physiological temperature range (Figure 6, shaded regions) results in a non-linear change in temperature throughout the walking bout. The range estimated using the net work (plus/minus the standard deviation) contains the experimental temperature, indicating that if the net work is close to zero, the experimental temperature could by generated by physiological factors alone.

#### 3.2.3 Estimated time to reach a first degree burn threshold

**FIGURE 7:**
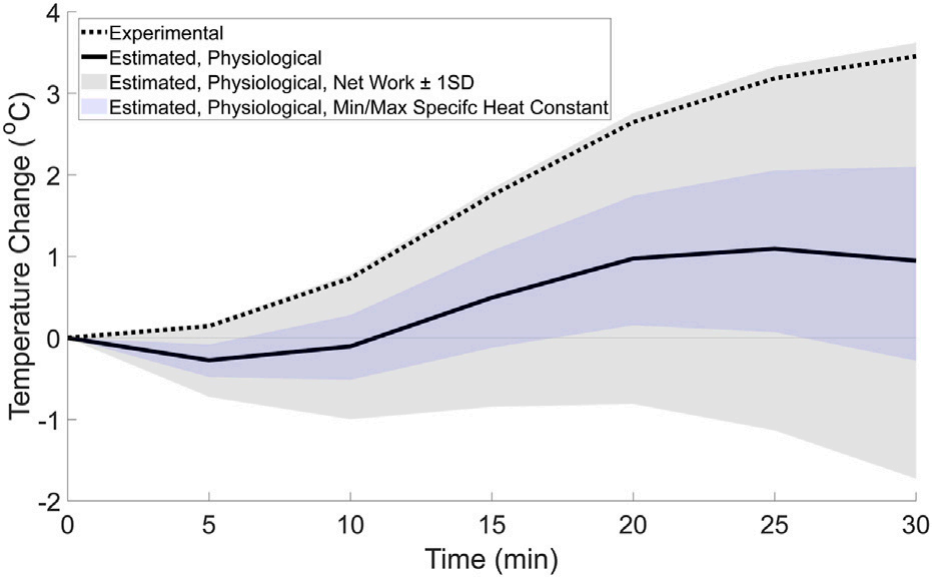
Estimated temperature change from physiological factors (solid line) based on the difference between the whole foot’s experimental temperature change, and the estimated mechanical factor’s contribution. For comparison, the range of possible temperatures from the physiological factors was calculated using the estimated upper and lower bounds for the mechanical factors based on the net work ±1 standard deviation (gray shaded region) and the minimum and maximum specific heat constants (blue shaded region). The whole foot’s experimental temperature change (dotted line) from the weighted average equation (Equation 1) is also displayed in the figure.

The walking time required for the foot to surpass the first-degree burn threshold of 43.3 °C (Johnson et al., 1974; Moritz and Henriques, 1947) is approximately 160 min (Figure 7, solid black line). However, the time to surpass the threshold may be as little as 77.5 min (Figure 7, gray shaded region), depending on the net work done by the foot and tissue distribution within the foot. For context, walking for 77.5 to approximately 160 min at 1.25 m/s would result in an individual walking between approximately 5.8 and 12 km.

## 4 Discussion

This study combined experimental and theoretical methods to generate novel hypotheses regarding how mechanical and physiological factors may interact affect temperature changes at the foot during a 30-min walk. The results of this study found that a non-linear model fit the experimental data better than a linear model (Figure 4; Table 3), suggesting that both mechanical and physiological factors may contribute to temperature responses at the foot during a walk. To further support the evidence that both factors contribute to the temperature response, a theoretical estimation of the relative contributions to the experimental temperature change was explored. This theoretical estimation (Figure 5) suggested that the mechanical factors and foot work account for approximately 2.5 °C of the experimental temperature change (total temperature change: 3.50 °C) during the walk. Therefore, the mechanical factors may explain a portion, but not all, of the experimental temperature change. The temperature difference between the experimental data and the estimated mechanical factors’ contribution (temperature change of 1.0 °C), as well as the non-linear trend, is likely attributable to the physiological factors (Figure 6). We suspect that these physiological factors would include contributions such as skin blood flow via the capillaries and AVAs, as well as cooling mechanisms such as sweat evaporation, conduction, and convection. Overall, the combined experimental and theoretical results suggest there is a complex interaction of mechanical and physiological factors at the foot such that both factors potentially underlying the foot’s temperature changes during walking. We envision that our study provides a new framework to study the mechanical and physiological contributions of foot temperature regulation, which may be important for future studies aimed to understand mechanisms of impaired temperature regulation in populations at risk for skin complications (e.g., diabetes).

Within this study, the whole foot temperature was found to increase 3.50 ± 2.38 °C on average. This is at the lower end, but still comparable to previous literature that has investigated the foot temperature during walking and found the temperature increased between approximately 2 and 12 °C when walking for between 10 and 45 min (Yavuz et al., 2014; Reddy et al., 2017; Ning et al., 2022). Differences in the foot temperature change in this study and previous literature may be due to the methods used. For example, in this study, thermal photos were taken throughout the walk in this study, requiring the shoes to be removed for a brief period of time during the walk. In the previous literature, the foot temperature was only measured before and after the walk was completed (Yavuz et al., 2014), or used in shoe temperature sensors which recorded temperature throughout (Reddy et al., 2017; Ning et al., 2022), therefore not requiring shoe removal during the walk. In addition, the speed and shoe choices for the walk were different between this study and others. In this study, participants wore their own shoes and walked at 1.25 m/s, rather than walking at approximately 1.4 m/s in standardized shoes (Ning et al., 2022) or at 0.9 m/s while barefoot (Yavuz et al., 2014). Therefore, these differences in methods may explain why the temperature change in this study is at the lower end of the previous literature’s temperature change.

The theoretical thermodynamics estimation was repeated with the foot net work (plus and minus one standard deviation), and the minimum and maximum specific heat capacity constants (Figure 5, shaded regions) to explore whether mechanical factors alone may explain the experimental temperature change. Interestingly, the experimental temperature change falls within the range of potential temperatures calculated using the foot net work plus and minus one standard deviation, but not the range of temperatures estimated using the minimum and maximum specific heat constants. And while this overlap would normally indicate that the mechanical factors alone could potentially generate the experimental temperature data, this result seems unlikely. For the mechanical factors to create a non-linear temperature change, the foot work, and resulting heat and temperature response, would need to vary throughout the walk. However, because the individuals in this study walked at the same speed throughout the 30-min walk, it is unlikely that the foot work, generated heat, and resulting skin temperature response would change by a large magnitude from stride to stride. Therefore, it seems unlikely that the mechanical factors alone would be expected to result in the non-linear experimental data found here.

Instead, the non-linear trend within the experimental data is likely due to physiological factors (Figure 6). A previous study found that the skin temperature during a run followed an S-shaped curve, initially decreasing, then increasing and plateauing (Tanda, 2015). Because the regions of the body studied previously (e.g., thigh, calf, trunk) were likely not exposed to high magnitude external mechanical factors, the temperature change was postulated to be due to physiological factors such as blood flow in the capillaries and AVAs. In the current study, not only does the experimental temperature follow a similar S-shaped trend where the temperature initially decreases, then increases and plateaus, but this trend can only be explained by the physiological contribution. Because the mechanical contribution from the foot work when walking at a constant speed is expected to be linear, the non-linearity can only be accounted for by variances in physiological factors during activity. Previous literature has suggested that there is a temporal variance in blood flow to the skin via the capillaries and AVAs which is likely driven by the core temperature thermoregulation (Johnson and Rowell, 1975; Kellogg et al., 1991a; Kellogg et al., 1991b; Johnson et al., 2010; Kenney and Johnson, 1992) or to regulate the temperature at the extremities during cold exposure (Singh et al., 2024). However, the role of AVAs at the feet during activity may be individual specific, as a validated model has suggested that a cold induced vasodilatory response may not occur at the feet in all individuals (Singh et al., 2024). In addition, cooling mechanisms such as sweat production and evaporation (Arens and Zhang, 2006; Machado-Moreira et al., 2008; Baker, 2019), conduction (Bornmyr et al., 1997; Arens and Zhang, 2006), and convection (Arens and Zhang, 2006; De Dear et al., 1997; Oguro et al., 2002) appear to have temporal variances which influence their effectiveness throughout the activity. Thus, the non-linearity found in the experimental data is likely related to the physiological factors, indicating that physiology must be considered in tandem with the mechanical factors when attempting to understand foot temperature responses.

Additional support that mechanics and physiology likely both contribute to foot temperature changes was suggested by estimating the time to surpass the first degree burn threshold of 43.3 °C with the theoretical thermodynamics model (Figure 7). Our theoretical estimates, depending on the assumptions used, ranged between approximately 77.5 and 160 min. When considering this range of times in the context of physical activity, it seems unlikely that a healthy individual would surpass a threshold where first degree burns may begin to develop after walking between approximately 5.8 and 12 km. As an anecdote, some individuals participate in long distance walking events where they walk farther than 20 km in a single day (Kay and Moxham, 1996; Crust et al., 2011; Yang et al., 2018). Therefore, when considered in the context of achievable physical activity distances and durations, mechanical factors alone are unlikely to drive foot temperature responses during activity. Instead, this theoretical calculation supports the idea that physiological factors provide thermoregulation of the skin temperature at the feet during activities like a long-distance walk.

There are several limitations with our experiment, theoretical calculations, and the conclusions. First, the experimental data consisted of a single thermal photo of the plantar surface of the foot taken every 5 minutes during the walk. The temporal resolution of the experimental data, and therefore the model fitting done in this study, is limited by the low resolution of the data. However, because the non-linear model fit the experimental data better even with this limited temporal resolution, the conclusions that both mechanical and physiological factors contribute to foot temperature would likely be strengthened by increasing the data’s temporal resolution. Additionally, this study focused only on the plantar surface, as this portion of the foot experiences the greatest mechanical factors and to minimize the pause time between 5-min walk periods. However, other aspects of the foot may also be important to assess to gain a more thorough understanding of the foot’s temperature response. Second, notable assumptions were required for the theoretical thermodynamics’ framework, including using average values based on the literature for the net work done by the foot, foot mass and tissue distribution, and number of strides taken during the 30-min walk. In addition, the theoretical framework assumes that the net work done by the foot does not vary from step to step, and that the only external mechanical contribution is from the dissipated energy which is transformed into heat and temperature changes. Instead, some energy is likely transformed into other forms such as sound (Oliveira et al., 2022), energy that is stored in the foot or transferred more proximally through multi-articular muscles (Honert and Zelik, 2016), or lost through conduction or convection throughout the walk. Therefore, the theoretical temperature change from mechanical factors and work done by the foot (Figure 5, solid line) likely represents a maximum value for temperature change for a single individual. Instead, the actual temperature change from mechanical factors may be slightly lower during a 30-min walk, as some of the dissipated energy is transformed into other forms besides heat or is lost through cooling mechanisms. Additionally, recent developments in simulation and thermal image analysis (Singh, 2022b) have suggested blood perfusion may be extracted using advanced methods (e.g., finite element analysis combined with thermal images). Future studies that integrate such emerging techniques may be valuable to further parse out the foot’s blood perfusion from thermal images. Finally, by assuming that only mechanical and physiological factors contribute to temperature changes at the foot, other sources of temperature change (e.g., environmental factors) are likely neglected in the conclusions here. Even with the highlighted limitations, this research indicates that there are likely several mechanisms (e.g., foot mechanics, physiology) contribute to the foot’s temperature change during activity, and that the relationship between skin temperature and activity is more complex than previously understood by studying potential mechanisms in isolation.

The results of this study emphasize the importance of investigating mechanical and physiological factors simultaneously to generate comprehensive hypotheses of temperature regulation. Such considerations may be important for future clinical studies aimed at identifying factors that lead to impaired temperature responses. As such, future research could be done to study mechanical and physiological factors in various populations with altered mechanics or physiology (e.g., peripheral arterial disease (Gatt et al., 2018; Meneses et al., 2018; Scott-Pandorf et al., 2007), diabetes (Martin, 1953; Moorhouse et al., 1966; DiLiberto et al., 2015), aging (Thijssen et al., 2016; Krupenevich et al., 2021; Pol et al., 2021)). For example, reducing blood flow to the limb through occlusion during activity in healthy adults may be able to isolate the influence of mechanical factors on the foot’s temperature. Similarly, comparing skin temperature changes during walking in individuals with and without vascular impairments (e.g., peripheral arterial disease (Gatt et al., 2018; Meneses et al., 2018), diabetes (Martin, 1953; Moorhouse et al., 1966), aging (Thijssen et al., 2016)) may be able to tease out the influence of skin blood flow and physiology on foot temperature. Altogether, our study provides new hypotheses regarding the interaction between mechanical and physiological factors involved in foot temperature regulation, which may provide a framework to study populations at risk for foot complications due to impaired temperature regulation.

## Data Availability

The raw data supporting the conclusions of this article will be made available by the authors, without undue reservation.
