# Supplementary material for: Foot temperature responses during walking: a theoretical estimation of mechanical and physiological factors
Source: Front Bioeng Biotechnol. 2025 Nov 20;13:1628846. doi: 10.3389/fbioe.2025.1628846 (PMC12675384; doi:10.3389/fbioe.2025.1628846)
Supplement: Supplementary file 1 [file Supplementaryfile1.docx]

1. **Supplementary information**

**Specific heat constant weighted average calculation for the foot**

The specific heat constant relates how much heat must be input to a unit of material to increase the temperature one degree. This constant will vary based on the object of interest’s material properties. Therefore, because the foot is primarily composed of five different tissues, including muscle, adipose, blood, bone, and skin, the specific heat constant of the foot can be estimated using a weighted average calculation (Eq. 5). In this case, the weighting factor was based on the mass of each tissue type (*m_muscle_, m_adipose_, m_blood_, m_bone_, m_skin_*) relative to the mass of the whole foot (*m_foot_*) (Taylor et al., 2014). The individual tissue weighting factors were multiplied by their respective specific heat constants (*c_muscle_, c_adipose_, c_blood_, c_bone_, c_skin_*) (Xu et al., 2023) and then these terms were summed to estimate the whole foot specific heat constant (*c_foot_*).

$$c_{foot}=\frac{m_{muscle}*c_{muscle}+m_{adipose}*c_{adipose}+m_{blood}*c_{blood}+m_{bone}*c_{bone}+m_{skin}*c_{skin}}{m_{foot}}$$

**(SEq. 1)**

The estimated specific heat constant for the whole foot was then used in Eq. 2 to estimate the change in temperature for the whole foot in response to the net work done during a single step.
